# USA stockpiling of remdesivir: How should the world respond?

**DOI:** 10.2217/cer-2020-0174

**Published:** 2020-12-04

**Authors:** Dalia Dawoud, Kalipso Chalkidou, Richard Sullivan, Francis J Ruiz, Amanda Adler

**Affiliations:** ^1^Faculty of Pharmacy, Cairo University, Cairo, Egypt; ^2^Global Health Policy & Center for Global Development, London, UK; ^3^Practice in Global Health, Imperial College London, London, UK; ^4^Cancer & Global Health, King’s College London, London, UK; ^5^Center for Global Development, Imperial College London, London, UK; ^6^NICE Technology Appraisal Committee B, University of Oxford, Oxford, UK

**Keywords:** antiviral, coronavirus, COVID-19, dexamethasone, remdesivir, SARS-CoV-2, stockpiling

## Abstract

The race to find an effective treatment for coronavirus disease 2019 (COVID-19) is still on, with only two treatment options currently authorized for emergency use and/or recommended for patients hospitalized with severe respiratory symptoms: low-dose dexamethasone and remdesivir. The USA decision to stockpile the latter has resulted in widespread condemnation and in similar action being taken by some other countries. In this commentary we discuss whether stockpiling remdesivir is justified in light of the currently available evidence.

As the number of coronavirus disease 2019 (COVID-19) cases continues to rise across the globe and passes 35 million confirmed cases, countries continue to race against the clock to find treatments and protective vaccines while battling a looming second wave [1].

In the absence of robust national pandemic preparedness strategies, many healthcare decision-makers have resorted to drastic and costly nonpharmaceutical measures known to control the spread of pandemic respiratory viruses [2]. However, the high basic reproduction number and case fatality rate of this novel coronavirus in older adults with underlying health conditions, has challenged responses even in high-income settings [3]. This has led to very long periods of national lockdown, closure of schools, travel restrictions and to the shutdown of many vital economic activities. While protective vaccines are developed, researchers around the world have focused efforts on finding effective treatments, with drug repurposing featuring as the fastest, most viable and least costly option [4].

## Drug repurposing

Drug candidates identified for repurposing as COVID-19 treatments were chosen based on historical evidence of activity against coronaviruses and similar RNA viruses, either *in vitro* or during earlier outbreaks [5]. The first two candidates that have shown early promise in randomized controlled trials were low-dose dexamethasone and remdesivir [6,7], though the benefit of the latter was less clear cut as discussed below. Aside from having very different mechanisms of action, these two drugs also differ in their sequencing in treating COVID-19, mode of administration, relative efficacy compared with the current standard of care and, most importantly from a resource allocation perspective, price.

Dexamethasone, a corticosteroid, has been used since the early 1960s to treat a wide range of conditions, such as rheumatoid arthritis and asthma. It is a generic drug that is currently available in both oral and injectable forms. The regimen trialled in hospitalized COVID-19 patients is 6 mg once daily for 10 days administered intravenously (iv.) [7]. The results from the RECOVERY trial showed that, compared with usual care, 482 patients (22.9%) in the low-dose dexamethasone plus usual care group and 1110 patients (25.7%) in the usual care group died within 28 days (age-adjusted rate ratio [RR] = 0.83; 95% CI: 0.75–0.93) [7]. In subgroup analysis, dexamethasone reduced 28-day mortality by a third in ventilated patients (RR = 0.65 [95% CI: 0.48–0.88]) and by one fifth in other patients receiving oxygen only (RR = 0.80 [95% CI: 0.67–0.96]) [7]. The latter arm included patients on noninvasive mechanical ventilation including high flow oxygen not readily available outside high-income countries’ hospital settings. This mortality benefit has led to optimism about the potential of not only managing severe COVID-19 and reducing its mortality, but also doing so with an affordable and widely accessible treatment option that will not deplete healthcare budgets across the globe – at least for those countries with sufficient high dependency and intensive care facilities [8]. Dexamethasone wholesale acquisition cost for a 10-day course of once-daily administration of 6 mg tablet is approximately $15 in the US [9].

On the other hand, remdesivir is a branded drug manufactured by Gilead Sciences which was originally developed for hepatitis C and Ebola [10]. The regimen trialled in hospitalized COVID-19 patients was iv. administration of 200 mg on day 1 followed by 100 mg on days 2–10 [6]. The pivotal trial of remdesivir plus usual care, compared with usual care alone, was a company-sponsored, randomized trial, the results of which showed a reduction of 4 days in median recovery time (remdesivir plus usual care: 11 days [95% CI: 9–12 days], usual care alone: 15 days [95% CI: 13–19 days]) and in 14-day mortality (hazard ratio [HR]: 0.70; 95% CI: 0.47–1.04) [6]. The trial was criticized for a change in its primary end point (with only about 15% of patients having their outcome determined at the specified 28 days) and early termination [11]. Another randomized placebo-controlled trial of remdesivir, however, showed that its use was not associated with a difference in time to clinical improvement (HR: 1.23; 95% CI: 0.87–1.75) [12]. This trial was also prematurely stopped for lack of patients to recruit as the outbreak in China was being controlled. Both studies showed that the effect of remdesivir on mortality is uncertain. This has also been the conclusion of a systematic review and network meta-analysis that compared all available treatments for COVID-19 [13]. This uncertainty around its impact on mortality has been reflected in the USA Institute of Clinical and Economic Review (ICER)’s estimates of the fair or value-based price (VBP) benchmark of remdesivir [9]. Before the announcement of the RECOVERY trial results, ICER initially estimated to this VBP to be US$4450 for a full course of treatment, assuming a benefit on mortality based on the mean estimate from its pivotal trial (HR = 0.7). However, following the announcement of the low-dose dexamethasone results in June 2020, it became clear that this ‘fair’ price should be revised. In response, ICER released its updated estimate that took into account the RECOVERY trial results for low-dose dexamethasone, on 24 June and updated its VBP benchmark to a maximum of US$2800, which is almost half the original estimate (US$4450), under best case scenario conditions that assumes a mortality benefit [9]. Of note, ICER calculated this benchmark based on a cost–effectiveness threshold of US$50,000 per quality-adjusted life-year gained, a value that is orders of magnitude higher than what would be applied in most countries around the world. Gilead Sciences has priced remdesivir at US$2340 for 5-day course for governments in developed countries [14].

## Stockpiling of remdesivir

Despite the research findings detailed above, the USA decision to stockpile remdesivir, by buying over 500,000 doses which represented all of the manufacturing company’s production for July and 90% of August and September production, has come as a surprise to many [15]. This move has been forecasted to deprive the UK, Europe and many other countries from access to this treatment for the 3 months to September 2020 [15]. It has been met by anger and scepticism around the world and prompted Gilead Sciences to issue a statement noting its intention to increase its manufacturing capacity to ensure adequate supply.

We argue that, based on the accumulating evidence, countries should not be overly concerned about this decision or feel compelled to do the same. So far, no study has demonstrated a reduction in hospital stay or mortality when using remdesivir, including the WHO SOLIDARITY trial which recently concluded that remdesivir has ‘little or no effect on hospitalized COVID-19 (patients), as indicated by overall mortality, initiation of ventilation and duration of hospital stay.’ [16]. This means in terms of cost–effectiveness and based on back-of-envelope calculations, remdesivir would be a ‘dominated option’ in patients who can instead be prescribed low-dose dexamethasone. There is also concern regarding possible link to liver damage in COVID-19 patients [17].

Furthermore, remdesivir has not shown benefit in any other group of hospitalized COVID-19 patients. In a possible bid to demonstrate better value, Gilead Sciences started assessing its potential for use in the earlier stages of the symptomatic phase of COVID-19, in an easy-to-administer form (inhalation) as opposed to intravenous infusion as currently used [18].

Thus, we believe (and others agree) that payers should not consider stockpiling remdesivir to avoid repeating the mistakes made with oseltamivir and zanamivir during flu outbreaks [11]. If remdesivir proves to be a clinically and cost-effective in any other group of patients, but availability is limited, the UK and other high-income countries could, if need be, secure supplies of remdesivir through a compulsory license. This compulsory license overrides the patent rights of Gilead Sciences and allows buying from generic manufacturers in countries like Bangladesh, Egypt and India, where remdesivir production has already started and its price has been set at almost 80% of that in the USA [19].

## Conclusion

Given the above, we should resist suspending the application of basic principles underlying good evidence-informed judgements. The USA decision to stockpile the drug at a higher-than merited price ignores potentially high opportunity costs for the health of its own population. The rest of the world should not feel compelled to do the same and should choose wisely.

The USA decision to stockpile remdesivir sets a bad precedent in relation to issues of access for any upcoming vaccines and other COVID-19 supplies at a time when global cooperation is needed. This action will create black markets for the treatments, a rise in the supply of substandard and falsified medicines and profiteering. But most pernicious of all, it will lead to misery for families of ill people, especially in poorer countries that lack universal health coverage, who will have to pay for these treatments themselves.
